# Potential Roles of Serum Caveolin-3 Levels in Patients with Atrial Fibrillation

**DOI:** 10.3389/fnagi.2017.00090

**Published:** 2017-04-04

**Authors:** Ling-Yue Sun, Xiang Qu, Ling-Zhi Chen, Gao-Shu Zheng, Xin-Lei Wu, Xing-Xing Chen, Wei-Jian Huang, Hao Zhou

**Affiliations:** ^1^Department of Cardiology, The First Affiliated Hospital of Wenzhou Medical UniversityZhejiang, China; ^2^Department of Clinical Laboratory, Wenzhou Central HospitalZhejiang, China

**Keywords:** atrial fibrillation, caveolin-3, left atrial diameter, heart failure

## Abstract

**Objective:** To explore serum caveolin-3 (Cav-3) levels in patients with atrial fibrillation (AF) and to evaluate the role of Cav-3 as a biomarker for AF and incident heart failure (HF).

**Methods:** Three hundred and five patients were enrolled in the study and divided into three groups: sinus rhythm (Group SR), paroxysmal AF (Group paAF), and persistent AF (Group peAF). Serum Cav-3 concentrations were measured by enzyme-linked immunosorbent assay at baseline. Clinical characteristics, and laboratory data were collected during hospitalization, and a follow-up of 12-months was carried out.

**Results:** Serum Cav-3 concentrations were significantly decreased on the Group SR and the highest concentrations of Cav-3 in patients were found on the Group peAF (516.7 ± 274.0 vs. 609.3 ± 287.0 vs. 688.3 ± 264.6 ng/L, *P* < 0.05). Left atrial diameter (LAD) in the Group peAF was significantly higher than in the Group paAF, and the Group SR had significantly lower LAD than the Group paAF and Group peAF. The risks of new-onset HF in the Group SR, Group paAF, and Group peAF were 8.1, 14.5, and 28.6%, respectively. There was a significant difference between the Group peAF and the other two groups. Serum Cav-3 concentrations were trisected in AF participants (lower tertile: ≤498, middle tertile: >498–703, upper tertile: ≥703). In further tertile studies, subjects in the lower tertile of Cav-3 concentrations were more likely to become paroxysmal AF and had much lower LAD (*P* < 0.05). And in the middle and upper tertiles, participants with AF tended to suffer from HF compared to the lower group (*P* < 0.05).

**Conclusion:** We provide evidence that Cav-3 has a significant meaning in AF patients. The levels of Cav-3 may be related to the LAD and new-onset HF.

## Introduction

Atrial fibrillation (AF) is the most common sustained cardiac arrhythmia, and it has been associated with an increased risk of stroke, heart failure (HF), cognitive dysfunction, impaired quality of life, as well as substantial health care costs, and eventually it contributes to an increased risk of cardiac and total mortality (Camm et al., 2010, 2012; January et al., 2014). Furthermore, it has shown that 8.8 million adults over 55 years had AF in 2010 and this number will more than double to 17.9 million by 2060 in the European Union (Krijthe et al., 2013). Considering the increased risks of diseases and mortality, expensive costs and increasing number of individuals associated with AF, there is a need for new biomarkers to assess AF.

Caveolae are small flask-like membrane invaginations and specialized microdomains in the sarcolemmal membrane of cardiomyocytes (Williams and Lisanti, 2004). Caveolae contain a variety of signaling proteins and cell surface receptors, including upstream entities [e.g., G protein-coupled receptors (Cohen et al., 2004; Ostrom and Insel, 2004), kinases (Prevostel et al., 2000) and phosphatases (Nilsson et al., 2006)] and downstream components (e.g., effector enzymes and ion channels; Vatta et al., 2006), which participate in membrane trafficking, sorting, transport, and signal transduction (Patel et al., 2008). And a multitude of studies focused on caveolin-3 (Cav-3) knockout (Woodman et al., 2002) and transgenic mice (Aravamudan et al., 2003; Patel et al., 2008) have highlighted the potential role of Cav-3 in cardiovascular pathophysiology. Cav-3 has been identified as a muscle-specific form of the caveolin family and plays a significant role in the pathophysiology of the heart (Williams and Lisanti, 2004), such as, cardiac arrhythmias (Vatta et al., 2006; Schilling et al., 2016), myocardial ischemia/reperfusion injury (Zhao et al., 2013), cardiac hypertrophy (Patel et al., 2008), and heart failure (Feiner et al., 2011).

Furthermore, recent evidence has suggested that caveolin-1 acting as an important anti-fibrotic signaling mediator plays a crucial role in the fibrosis of AF (Yi et al., 2014). However, little was known about the effects of Cav-3 on individuals with AF.

The purpose of this study attempts to investigate serum Cav-3 levels in AF patients, to explore the associations of Cav-3 with AF and incident HF, to evaluate the correlation of Cav-3 with echocardiographic parameters (left atrial diameter, LAD), to evaluate the role of Cav-3 as a biomarker for AF and incident HF. We hypothesized that Cav-3 might provide novel insights into the diagnosis and prognosis of AF.

## Materials and methods

### Study population

Between January and June 2015, our study enrolled 305 consecutive patients into the prospective, single-center project. According to standard clinical practice, all enrolled individuals who underwent 12-lead electrocardiograms (ECGs) and/or 24-h Holter monitoring. According to the diagnose and procedures of the Guidelines (Camm et al., 2010; January et al., 2014), individuals selected into our study were those patients diagnosed as AF. Patients were classified into 3 groups: those with sinus rhythm (Group SR), those with paroxysmal AF (Group paAF: self-terminating or with intervention within 7 days of onset), and those with persistent AF (Group peAF: lasting >7 days, and including long-standing persistent AF: continuous AF > 12 months in duration). Patient demographics, clinical diagnoses, laboratory results, brain CT scan, and echocardiogram examination were collected. Participants self-reported smoking and drinking status. Diagnosed diabetes mellitus (DM), hypertension and coronary artery disease (CAD) were defined as a self-reported physician diagnosis or current use of medications. Exclusion criteria were the following: cardiac valvular disease, stroke, congestive HF or reduced left ventricular ejection fraction (LVEF) <50%. Of the 356 subjects screened for inclusion, 305 patients met the inclusion criteria, and 51 individuals met 1 of the exclusion criteria. This study protocol was approved by the Institutional Review Board of the First Affiliated Hospital of Wenzhou Medical University, and all enrolled participants provided written informed consent.

### Measurement of Cav-3 and other exposure variables

Blood samples were collected from the peripheral veins on enrollment and anticoagulated with ethylenediaminetetraacetic acid. After centrifugation at 3,000 rpm for 10 min at 4°C, the serum was separated and stored at −80°C in aliquots until analysis. Samples were thawed only once before measurement. We measured Cav-3 in stored aliquots, using the Roche Cav-3 reagent kit, a sandwich immunoassay, performed on a Roche Elecsys 2010 Analyzer (Roche Diagnostics, Indianapolis, Indiana). Serum lipid concentrations, serum glucose, C-reactive protein (CRP), N-terminal probrain natriuretic peptide (NT pro-BNP) were determined with standardized protocols.

### Follow-up

All subjects were contacted via telephone, an outpatient clinic and hospitalization with follow-up every 3 months. At last follow-up, we collected the records of 12-lead ECGs and/or 24-h Holter monitoring, and echocardiogram evaluation for all participants. Major adverse events, including ischemic stroke, hospitalization, AF recurrence, HF, all-cause death, were recorded. AF recurrence was identified by symptoms with ECG documentation of an AF lasting <30 s on a 12-lead ECG, event recording, or Holter monitor recording. For those patients underwent catheter ablation, AF that occurred was not counted as recurrences during the first 2 months. HF was defined with the records based on decreased LVEF (<50%) and the clinical symptoms and signs (e.g., breathlessness, orthopnea, fatigue, ankle swelling, elevated jugular venous pressure, pulmonary crackles, and S3 gallop). Patients with NT-pro BNP values in between 100 and 400 pg/ml needed to be assessed clinically. These clinical signs and symptoms almost have required diuretics, inotropic support or vasodilator therapy.

### Statistical analysis

Normally distributed variables are expressed as mean ± standard deviation (SD) and compared using one-way analysis of variance. We further divided AF participants into trisection based on distribution of Cav-3. Two-sided *P* < 0.05 were considered to indicate statistical significance (SPSS Statistics ver. 20.0, IBM Corporation, Armonk, USA). Figures were made using GraphPad Prism 6.0 (GraphPad Software, La Jolla, CA).

## Results

### Characteristics of the study population

**Figure 1:**
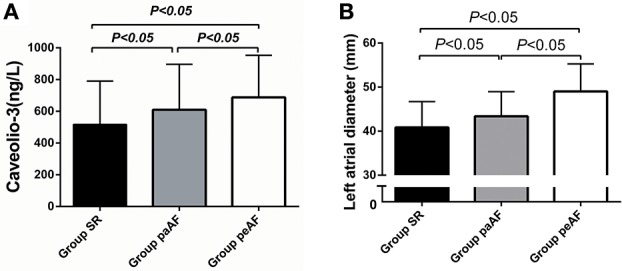
**Differences of Caveolio-3 concentrations (A)** and left atrial diameter **(B)** among three groups.

The demographic, clinical characteristics, and laboratory findings for all 305 patients enrolled in the present study are summed in Table 1. Totally, 124 patients with SR (age, 62.8 ± 11.9 years; sex, 34.7% female), 76 with paroxysmal AF (age, 65.8 ± 10.8 years; sex, 42.1% female), and 105 with persistent AF (age, 66.7 ± 10.8 years; sex, 35.2% female) were included. Participants in the Group peAF were older than those in the Group SR (*P* < 0.05). And the Group paAF had a much lower BMI than the other two groups (*P* < 0.05). Data from medical history (CAD, hypertension, DM, etc) and clinical parameters (NT-pro BNP, CRP, Creatinine clearance rate, etc) showed no significant differences among the three groups. Among the three groups, serum Cav-3 concentrations were significantly decreased on the Group SR and the highest concentrations of Cav-3 in patients were found on the Group peAF (516.7 ± 274.0 vs. 609.3 ± 287.0 vs. 688.3 ± 264.6 ng/L, *P* < 0.05) (Figure 1A). Similarly, regarding echocardiographic analysis, there were significant differences among the three groups in the LAD (Figure 1B). LAD in the Group peAF were larger than those in the Group paAF, and the Group SR demonstrated the lowest LAD among three groups (Figure 1B).

**Table 1:** **Baseline demographic and clinical characteristics of study participants**.

**Characteristics**	**Group SR**	**Group paAF**	**Group peAF**	***P*-value**
Participants, *n* (%)	124 (40.7)	76 (24.9)	105 (34.4)	
**DEMOGRAPHIC DATA**				
Age, years	62.8 ± 11.9	65.8 ± 10.8	66.7 ± 10.8	*P* < 0.05^&^
Female (%)	43 (34.7)	32 (42.1)	37 (35.2)	NS
BMI, kg/m^2^	25.4 ± 4.6	24.0 ± 3.0	25.2 ± 3.4	*P* < 0.05^#^^,^^*^
Duration of AF, years	–	2.6 ± 3.7	4.7 ± 5.2	*P* < 0.05^*^
**MEDICAL HISTORY**, ***n*** **(%)**				
CAD, *n* (%)	43 (34.7)	20 (26.3)	27 (25.6)	NS
Hypertension, *n* (%)	67 (54.0)	41 (53.9)	63 (60.0)	NS
DM, *n* (%)	24 (19.4)	18 (23.7)	25 (23.8)	NS
Smoking, *n* (%)	38 (30.6)	17 (22.4)	24 (22.6)	NS
Alcoholism, *n* (%)	28 (22.6)	13 (17.1)	21 (20.0)	NS
**LABORATORY MEASUREMENTS**				
Caveolio-3, ng/L	516.7 ± 274.0	609.3 ± 287.0	688.3 ± 264.6	*P* < 0.05
NT pro-BNP, pg/ml	183.2 ± 380.5	195.9 ± 239.0	278.2 ± 234.1	NS
Ccr, ml/min	84.4 ± 29.0	78.2 ± 27.3	78.1 ± 27.4	NS
LDL-C, mmol/l	2.6 ± 1.0	2.7 ± 1.1	2.9 ± 1.7	NS
CRP, mg/L	6.2 ± 10.4	9.4 ± 20.5	10.4 ± 20.8	NS
Serum glucose, mmol/L	5.2 ± 1.4	6.1 ± 4.6	5.5 ± 1.7	NS
**ECHOCARDIOGRAPHIC DATA**				
LAD, mm	40.8 ± 5.9	43.4 ± 5.6	49.0 ± 6.3	*P* < 0.05
LVEDD, mm	50.7 ± 7.5	49.8 ± 6.3	50.7 ± 6.5	NS
LVEF, (%)	62.3 ± 12.1	62.2 ± 8.8	60.9 ± 10.5	NS
CA, *n* (%)	0	30 (39.5)	37 (35.2)	NS^*^

*BMI, Body mass index; CAD, coronary artery disease; DM, Diabetes mellitus; Ccr, Creatinine clearance rate; LDL-C, low density lipoprotein-cholesterol; CRP, C-reactive protein; LAD, left atrial diameter; LVEDD, left ventricular end-diastolic dimension; LVEF, left ventricular ejection fraction; CA, catheter ablation*.

# *P < 0.05, P-value between the Group SR and the Group paAF*.

& *P < 0.05, P-value between the Group SR and the Group peAF*.

* *P < 0.05, P-value between the Group paAF and the Group peAF*.

*P < 0.05, overall*.

NS, no significance; NS^*^, no significance between the Group paAF and the Group peAF.

**Figure 2:**
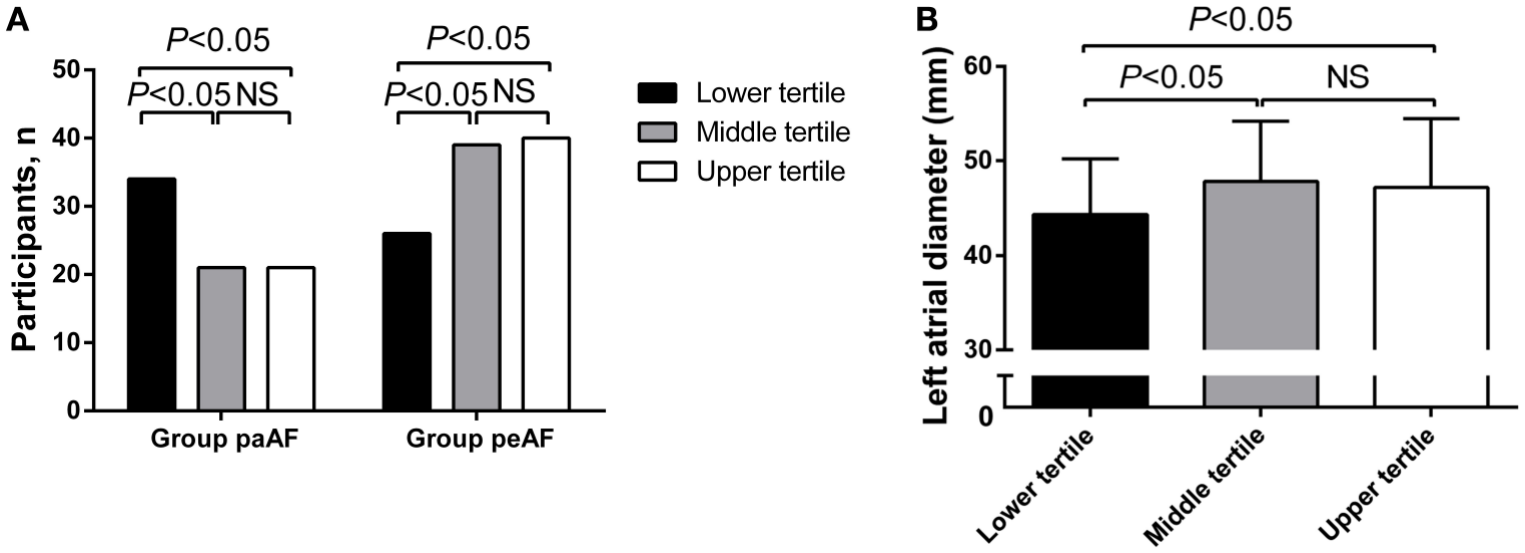
**Differences in number of participants (A)** and left atrial diameter **(B)** in the Group paAF and the Group peAF by tertile of circulating Cav-3 concentrations.

Furthermore, serum Cav-3 concentrations measured in AF participants were classified into trisection (lower tertile: ≤498, middle tertile: >498–703, upper tertile: ≥703) (Table 2). Participants with lower serum Cav-3 concentrations were more likely to become paroxysmal AF (*P* < 0.05) (Figure 2A). As presented in Table 2, the numbers of persistent AF patients were 39 and 40, respectively. In the middle and upper tertiles, the Group peAF had higher levels of Cav-3 than the Group paAF. In further study to evaluate the relationship between Cav-3 concentrations and echocardiographic parameters, subjects in the lower tertile of Cav-3 had lower LAD, compared to the other two groups (*P* < 0.05) (Figure 2B). And the middle and upper tertiles demonstrated no statistical differences between the Group paAF and the Group peAF. There were no significant differences of LAD between the middle tertile and upper tertile. In tertile analysis, no significant differences were observed between serum Cav-3 in medical history (CAD, hypertension, DM, etc), clinical parameters (NT-pro BNP, CRP, Creatinine clearance rate, etc) and echocardiographic measurements (left ventricular end-diastolic dimension and LVEF) among the three groups.

**Table 2:** **Demographics and Clinical Characteristics at Baseline by tertile of circulating Cav-3 concentrations in AF subjects**.

	**Caveolio-3 Level, ng/L**			***P*-value**
	**≤498**	**>498–703**	**≥703**	
Participants, *n* (%)	60	60	61	
paAF, *n* (%)	34 (56.7)	21 (35.0)	21 (34.4)	*P* < 0.05^#^^,^^&^
peAF, *n* (%)	26 (43.3)	39 (65.0)	40 (65.6)	*P* < 0.05^#^^,^^&^
**DEMOGRAPHIC DATA**				
Age, years	64.6 ± 10.2	67.3 ± 11.0	67.0 ± 11.1	NS
Female (%)	24 (40.0)	19 (31.7)	26 (43.3)	NS
BMI, kg/m^2^	24.7 ± 3.5	24.4 ± 3.0	25.0 ± 3.4	NS
Duration of AF, years	3.4 ± 4.9	4.3 ± 5.0	3.8 ± 4.2	NS
**MEDICAL HISTORY**, ***N*** **(%)**				
CAD, *n* (%)	14 (23.3)	16 (26.7)	17 (27.9)	NS
Hypertension, *n* (%)	30 (50.0)	35 (58.3)	39 (63.9)	NS
DM, *n* (%)	14 (23.3)	14 (23.3)	15 (24.6)	NS
Smoking, *n* (%)	13 (21.7)	17 (28.3)	11 (18.0)	NS
Alcoholism, *n* (%)	10 (16.7)	14 (23.3)	10 (16.4)	NS
**MEDICAL HISTORY, N (%)**				
NT pro-BNP, pg/ml	218.0 ± 228.7	256.9 ± 209.7	255.9 ± 274.6	NS
Ccr, ml/min	82.5 ± 26.7	75.6 ± 30.3	76.4 ± 24.5	NS
LDL-C, mmol/l	3.0 ± 2.2	2.6 ± 0.8	2.8 ± 1.0	NS
CRP, mg/L	7.5 ± 16.9	8.5 ± 18.5	13.6 ± 25.0	NS
Serum glucose, mmol/L	5.3 ± 1.1	6.1 ± 4.9	5.7 ± 2.4	NS
**ECHOCARDIOGRAPHIC DATA**				
LAD, mm	44.4 ± 5.9	47.8 ± 6.4	47.2 ± 7.2	*P* < 0.05^#^^,^^&^
LVEDD, mm	50.1 ± 6.0	50.2 ± 6.8	50.9 ± 6.3	NS
LVEF, (%)	61.9 ± 9.0	61.2 ± 10.0	61.4 ± 10.2	NS
CA, *n* (%)	27 (45.0)	19 (31.7)	21 (34.4)	NS

*BMI, Body mass index; CAD, coronary artery disease; DM, Diabetes mellitus; Ccr, Creatinine clearance rate; LDL-C, low density lipoprotein-cholesterol; CRP, C-reactive protein; LAD, left atrial diameter; LVEDD, left ventricular end-diastolic dimension; LVEF, left ventricular ejection fraction; CA, catheter ablation; NS, no significance*.

# *P < 0.05, P-value between the Group SR and the Group paAF*.

& *P < 0.05, P-value between the Group SR and the Group peAF*.

**Figure 3:**
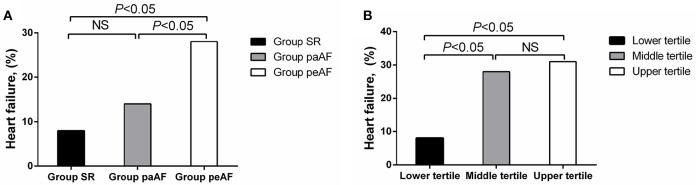
**Differences in number of incident heart failure in the Group SR, the Group paAF, and the Group peAF (A)**, and in the tertile of Cav-3 **(B)**.

During follow-up period of 12 months, 4 patients died, 4 suffered from stroke, 11 were with AF recurrence, 34 were readmitted, and 51 developed HF. There had no significant differences about all-cause death, stroke and hospitalization among the three groups, and no significant differences about AF recurrence between the Group paAF and the Group peAF. The risks of new-onset HF in the Group SR, Group paAF, and Group peAF were 8.1, 14.5, and 28.6%, respectively. Patients in the Group peAF had a much higher risk to develop new-onset HF than other two groups, while no differences were shown in the Group SR and the Group paAF (Figure 3A). With further tertile analysis of Cav-3, the lower tertile demonstrated the lowest risk of new-onset HF among the three groups and had a significant difference compared with the middle and upper tertiles (Figure 3B). Major adverse events were displayed in Tables 3, 4.

**Table 3:** **Major adverse events of study participants**.

	**Group SR *N* = 124**	**Group paAF *N* = 76**	**Group peAF *N* = 105**	***P*-value**
All-cause death, *n* (%)	0	2 (2.6)	2 (1.9)	NS
Hospitalization, *n* (%)	10 (8.1)	14 (18.4)	20 (19.0)	NS
Stroke, *n* (%)	0	1 (1.3)	3 (2.9)	NS
AF recurrence, *n* (%)	0	6 (7.9)	5 (4.8)	NS^*^
HF, *n* (%)	10 (8.1)	11 (14.5)	30 (28.6)	*P* < 0.05^#^^,^^*^

HF, heart failure; AF, atrial fibrillation; NS, no significance; NS^*^, no significance between the Group paAF and the Group peAF.

# *P < 0.05, P-value between the Group SR and the Group paAF*.

* *P < 0.05, P-value between the Group paAF and the Group peAF*.

*P < 0.05, overall*.

**Table 4:** **Major adverse events of AF participants by tertile of circulating Cav-3 concentrations in AF subjects**.

	**Caveolio-3 Level, ng/L**			***P*-value**
	**≤498 (60)**	**>498–703 (60)**	**≥703 (61)**	
All-cause death, *n* (%)	1 (1.7)	0	3 (4.9)	NS
Hospitalization, *n* (%)	10 (16.7)	16 (26.7)	8 (13.1)	NS
Stroke, *n* (%)	0	3 (5.0)	1 (1.6)	NS
AF recurrence, *n* (%)	4 (14.8)	4 (21.1)	3 (14.3)	NS
HF, *n* (%)	5 (8.3)	17 (28.3)	19 (31.1)	*P* < 0.05^#^^,^^&^

*HF, heart failure; AF, atrial fibrillation; NS, no significance*.

# *P < 0.05, P-value between the Group SR and the Group paAF*.

& *P < 0.05, P-value between the Group SR and the Group peAF*.

*P < 0.05, overall*.

## Discussion

There is a growing body of data within the literature that implicate Cav3 as a causative agent of a wide range of cardiovascular pathophysiologies (Vatta et al., 2006; Patel et al., 2008; Tsutsumi et al., 2008; Feiner et al., 2011; Zhao et al., 2013; Schilling et al., 2016). Of the above studies, the function of Cav-3 has been represented in animal models. There has lacked clinical data to confirm the effect of Cav-3 on heart disease, even less on AF. The major findings generated from our study are as follows: high Cav-3 had a significant relationship with AF participants compared with patients in SR. In addition, Cav-3 concentrations were much higher in the Group peAF than the Group paAF. It showed that concentrations of Cav-3 might be associated with the frequency and duration of AF. And moreover, our study demonstrated that levels of Cav-3 had a close relationship with LAD, a common and vital risk factor of AF. Regarding incident HF in AF subjects, Cav-3 concentrations had a certain association with the occurrence of HF.

Studies spanning more than several decades still reach no consensus on certain mechanisms of AF (Camm et al., 2010; January et al., 2014). It currently tends to acceptable that pathophysiological changes (Kistler et al., 2004; Schotten et al., 2011) and electrophysiological disorders (Haïssaguerre et al., 1998; Calkins et al., 2012) trigger for the onset of AF. Abnormal intracellular potassium and calcium handling, as well as ion channels, may play a role in AF. Similarly, atrial structural remodeling facilitates the initiation and perpetuation of AF. Perturbation of atrial contractile function also occurs within days of AF. Either pathophysiological changes or electrophysiological disorders may initiate the onset of AF, but both of them coexist in perpetuation of AF (Schotten et al., 2011). To our knowledge, Cav-3 takes a significant part in arrhythmias (Vatta et al., 2006; Schilling et al., 2016) and cardiac hypertrophy (Patel et al., 2008). Cav-3 plays a critical role in organizing signaling molecules (Cohen et al., 2004; Ostrom and Insel, 2004) and ion channels (Patel et al., 2008) involved in cardiac conduction and hypertrophy. Previous work demonstrates that Cav-3 overexpression in cardiomyocytes is essential for promoting the protective signaling in pathological cardiac hypertrophy (Markandeya et al., 2015). On the contrary, loss of Cav-3 expression is prone to induce a molecular program leading to cardiomyocytes hypertrophy and cardiomyopathy (Woodman et al., 2002). Considering that the crucial effect of Cav-3 on the heart, we harbor the idea that Cav-3 also has a close relationship with AF. Those findings observed from animal models are consistent with our clinical study. Similar findings are demonstrated in our study that Cav-3 concentrations are reduced in the patients with SR compared with those in AF patients. To the further extent, the Group paAF has higher levels of Cav-3 than the Group peAF. In tertile analysis, subjects in the Group paAF tend to have the lower levels of Cav-3. Similarly, the first tertile has lower LAD and lower occurrence of HF than the other two groups. Accordingly, we could speculate that Cav-3 has a certain relationship with the paroxysmal AF, LAD, and incident HF. However, when Cav-3 concentrations are beyond some specific value, we find no direct interaction between Cav-3 and AF, as well as LAD, occurrence of HF. Our study was limited by a small study population and short follow-ups. Further studies with subsequent follow-up are favored. Patients were recruited from a single university hospital. And there was missing Cav-3 sample collected at the last follow-ups. The pathophysiological role of Cav-3 in AF needs further studies.

The present study shows that serum Cav-3 levels are significantly elevated and have a significant meaning in AF patients. The levels of Cav-3 may be related to the LAD and new-onset HF.

## Author contributions

All authors (LS, XQ, LC, GZ, XW, XC, WH, and HZ) meet all 4 of the criteria: (1) Substantial contributions to the conception or design of the work; or the acquisition, analysis, or interpretation of data for the work. (2) Drafting the work or revising it critically for important intellectual content. (3) Final approval of the version to be published. (4) Agreement to be accountable for all aspects of the work in ensuring that questions related to the accuracy or integrity of any part of the work are appropriately investigated and resolved.

## Funding

This research was supported by the National Natural Science Foundation of China (81570364), and in part by the Science and Technology Planning Project of Wenzhou Science; Technology Bureau of Zhejiang Province of China (Y20150032 and Y20160118), the Foundation for the Program of Science and Technology Department of Zhejiang Province of China (2014C33166), and the Foundation for the Program of the Provincial Health Department of Zhejiang Province of China (2014KYA136).

### Conflict of interest statement

The authors declare that the research was conducted in the absence of any commercial or financial relationships that could be construed as a potential conflict of interest.
